# A dual electro-optical biosensor based on *Chlamydomonas reinhardtii* immobilised on paper-based nanomodified screen-printed electrodes for herbicide monitoring

**DOI:** 10.1186/s12951-021-00887-4

**Published:** 2021-05-17

**Authors:** Amina Antonacci, Raouia Attaallah, Fabiana Arduini, Aziz Amine, Maria Teresa Giardi, Viviana Scognamiglio

**Affiliations:** 1 Department of Chemical Sciences and Materials Technologies, Institute of Crystallography, National Research Council, Via Salaria km 29.300, Monterotondo, 00015 Rome, Italy; 2 Faculty of Sciences and Techniques, Hassan II University of Casablanca, Casablanca, Morocco; 3 Department of Chemical Science and Technologies, University of Rome “Tor Vergata”, Via della Ricerca Scientifica, 00133 Rome, Italy; 4 SENSE4MED, Via Renato Rascel 30, 00128 Rome, Italy; 5 Biosensor Srl, Via degli Olmetti 44, Formello, 00060 Rome, Italy

**Keywords:** Electro-optical transduction, Screen-printed electrodes, Paper-based biosensors, GRAS organisms, Eco-friendly

## Abstract

The indiscriminate use of herbicides in agriculture contributes to soil and water pollution, with important endangering consequences on the ecosystems. Among the available analytical systems, algal biosensors have demonstrated to be valid tools thanks to their high sensitivity, cost-effectiveness, and eco-design. Herein, we report the development of a dual electro-optical biosensor for herbicide monitoring, based on *Chlamydomonas reinhardtii* whole cells immobilised on paper-based screen-printed electrodes modified with carbon black nanomaterials. To this aim, a systematic study was performed for the selection and characterisation of a collection among 28 different genetic variants of the alga with difference response behaviour towards diverse herbicide classes. Thus, CC125 strain was exploited as case study for the study of the analytical parameters. The biosensor was tested in standard solutions and real samples, providing high sensitivity (detection limit in the pico/nanomolar), high repeatability (RSD of 5% with n = 100), long lasting working (10 h) and storage stability (3 weeks), any interference in the presence of heavy metals and insecticides, and low matrix effect in drinking water and moderate effect in surface one.

**Supplementary Information:**

The online version contains supplementary material available at 10.1186/s12951-021-00887-4.

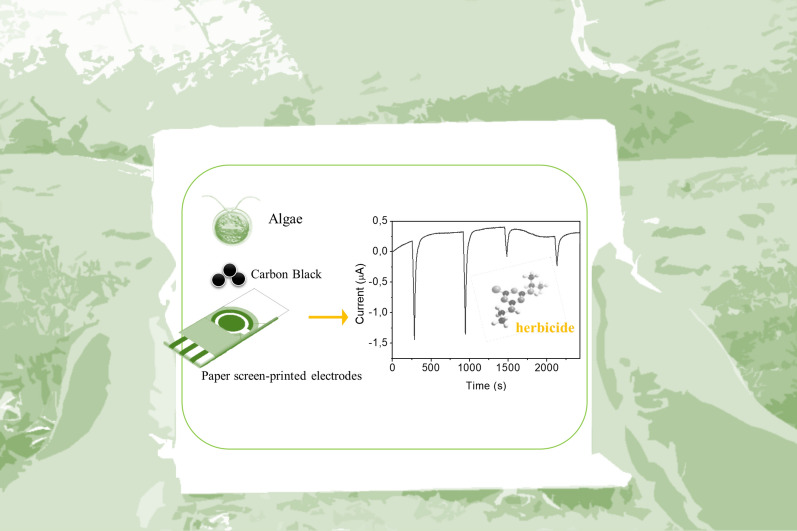

## Introduction

In the last decades, pollution of soil and water from toxic chemicals as pesticides caused general concerns for the resulting harmful effects on the ecosystems and human health. Approximately, 2 million tonnes of pesticides are annually exploited worldwide with a total sale over the period 2011–2018 of around 360.000 tonnes per year in the EU. The major pesticide groups which recorded the highest sales volumes have been “fungicides and bactericides” and “herbicides, haulm destructors and moss killers” [13]. In particular, herbicides belonging to the triazine and ureic classes are largely exploited in agriculture to remove grasses and weeds for enhancing crop yield and thus ensuring global food security. Although their usage is beneficial for crop production, extensive use of herbicides causes important detriment on animals and ecosystems, due to their bio-magnification and persistent nature. This urgent issue struggled the development of new technologies, in support of the conventional ones, for the monitoring of these compounds in the environment. The last trends on the design of high-throughput platforms exploiting algae seems to be a versatile solution for the fabrication of biosensors [2]. Indeed, these photosynthetic microorganisms are very sensitive to specific environmental changes, enabling the detection of ultra-traces of pollutants. In particular, algae-based biosensors represent intriguing devices for photosynthetic herbicide detection (triazine and ureic classes). Their mechanism of action is strictly related to the fluorescence of the chlorophyll *a* and its modulations in the presence of xenobiotic compounds. The strong potential of algae-based biosensors relies on reliable monitoring of these intracellular variations in real time and in situ, basically by two main transduction methods, (i) optical (i.e., fluorescence induction curves), and (ii) electrochemical (i.e., amperometry), both in a herbicide concentration-dependent manner [2]. The use of algae as biorecognition element tightly fits with biosensor requirements, in particular concerning the sensitivity able to meet the MRL of EU Directives for drinking and surface waters (EU Directive 98/83/EC, EU Dir. 2013/39/EC).

Several promising studies from the scientific literature highlight the appealing and versatile technology of algae-based biosensors, thanks to many advantages, including very high sensitivity in the pico/nanomolar range, fast analysis (15 min), high operational stability (10–12 h), medium storage stability (3 weeks at room temperature under light), high repeatability, optimal conjunction of algae with nanomaterials e.g., carbon black nanoparticles and smart materials e.g., paper substrate, simple integration into portable sensing prototypes also in dual transduction system i.e., optical and electrochemical, the availability of interchangeable bioreceptors (able to recognise many toxic chemicals as herbicides and chemical weapons), reversible mechanisms of herbicide detection (repeated analysis of the target through washes with buffer), and no need for sample pre-treatment [8, 12, 14, 24].

Many biosensors reported in literature, using photosynthetic recognition elements, specifically exploit the inhibition of PSII photochemical reaction in the presence of different toxic compounds. Several studies report the design of optical and electrochemical biosensors exploiting both whole microalgae cells and their sub-components as thylakoid membrane and extracted PSII or reaction centres. Nevertheless, these analytical systems suffer from some drawbacks which hinder their practical use in-the-field, as low selectivity as well as low yield of algae immobilisation protocol, which results in a low repeatability in the preparation of algae-based sensors [2]. With the intent to contribute in coping with these main drawbacks, we propose an efficient approach to test whole cells of *C. reinhardtii* as a good biocomponent for the development of an eco-designed dual transduction algal biosensor for herbicides detection, exploiting the green photosynthetic alga *Chlamydomonas reinhardtii* immobilised on paper-based screen-printed electrodes modified with carbon black nanomaterials (pCB-SPEs). The novelty of this study relies on: (i) the systematic study of a huge collection of *C. reinhardtii* genetic variants, performed for the first time to widen the availability of smart bioreceptors for herbicide detection among different classes from triazine to ureas, and (ii) the immobilisation of algae whole cells on nanomodified electrodes scree-printed on paper as substrate. This choice also encompasses the fundamentals of sustainability of the 2030 Agenda for Sustainable Development [1, 5, 6, 9], because of the eco-friendly nature of both paper substrate and the algae, recognised by the Food and Drug Administration (FDA) as a Generally Recognized as Safe (GRAS) organism [25].

## Materials and methods

### Growth conditions of the algal liquid cultures

All the strains of *C. reinhardtii* were maintained under continuous light (50 µmol photons m^−2^ s^−1^), at 25 °C, and on tris-acetate-phosphate (TAP) agar culture medium plates. Prior to each experiment, each strain was collected with a sterile loop and used to inoculate stock culture made in 100 mL flasks with 50 mL of liquid TAP closed with bacteriological cotton. Flasks were placed into an orbital shaker at 25 °C, stirring at 150 rpm and under continuous light (50 µmol photons m^−2^ s^−1^). All the materials and media used for the inoculation were sterilized in the autoclave at 120 °C and 1 bar pressure for 20 min. The inoculation was performed under the biological fume hoods to avoid any contamination.

After 72 h from inoculation the optical density of each strain culture was checked by spectrophotometer analysis at a 750 nm wavelength in TAP medium. After this measurement, the algae cultures were diluted in TAP to an optical density of 0.15 O.D._750_ in a final volume of 200 mL. Then the refreshed cultures were mixed on an orbital shaker under the same conditions above reported for all the period of the physiological characterisation.

### Algae physiological characterization

All experiments were performed under continuous light (50 µmol photons m^−2^ s^−1^) and agitation (150 rpm) at 25 °C, starting with cell cultures in early mid-exponential growth phase, with Abs_750_ 0.5 O.D., 10^6^ cells/mL, and 5 µg/mL chlorophyll content. Cell culture growth was spectrophotometrically evaluated measuring the absorbance (O.D.) at 750 nm wavelength. Cell number was quantified using a Bio-Rad TC-10 automated counter (Hemel Hempstead, UK), using a 10 µL-volume cell counting slide. Pigment content was spectrophotometrically measured by quantifying the absorbance (O.D.) of the chlorophylls *a* and *b* at 652 nm wavelength, once extracted with 80% acetone. Chlorophyll extraction: 1 mL of cell culture was harvested and centrifuged at 20,000*g* for 5 min to destroy all the phospholipid membranes and preserve the pigments. 800 µL of the supernatant was removed and the remaining pellet and 200 µL supernatant was diluted by adding 800 µL of 100% aqueous acetone. The solution is vortexed for 2 min in dark and then centrifuged for 3 min. The total of chlorophyll content was determined spectrophotometrically measuring the absorbance at 652 nm (80% acetone as blank). The calculation of the total chlorophyll concentration expressed as µg/mL was performed by the equation: (O.D._652_ × 1000)/34.5 [7, 28]. The photosynthetic profile was assessed by the chlorophyll *a* fluorescence induction (Kautsky) curves, recorded with a Plant Efficiency Analyzer (PEA) at room temperature after 10 min of dark adaptation and with a 5 s saturating pulse excitation light (3500 µmol photons m^−2^ s^−1^) using an array of six red light emitting diodes (650 nm peak). The measurement was carried out in a small flask containing 5 mL culture in two steps: (i) *in the dark* to ensures the removal of energy motor of photosynthesis (photons) and discharge the photosynthetic chain from electrons; then the reaction centres are all ready for the acceptance of new electrons and the photosynthetic machinery is actually in a basal state of zero fluorescence (F_0_), and (ii) *under light* to provide saturating light flash, thus inducing a reduction of all reaction centres (Q_A_) and obtaining a maximum fluorescence value (F_M_). The excess of light energy, uncollected by PSII, takes different paths, including heat dissipation and fluorescence emission, according to a particular kinetics; then the electron transfer chain is switched on and reaches a steady state.

Kautsky curves or OJIP curves are defined by a polyphasic fluorescence rise in the time, with O as the minimal dark-acclimated fluorescence level (indicating that all Q_A_ are oxidised) and P as the maximal level (indicating that all PSII quinone acceptors are fully reduced). The difference in the fluorescence signal of these distinct states helps evaluating the PSII functionality through the following parameters calculated by the fluorimeter:

- F_0_ or fluorescence in initial state: minimum fluorescence intensity in the state acclimated to the darkness, recorded when all PSII reaction centres are open (oxidized quinones).
- F_M_ or maximum fluorescence: maximum fluorescence intensity reached after 10 min of darkness and a subsequent saturating light pulse, recorded when all reaction centres of the PSII are closed (reduced) [26].
- F_V_/F_M_: maximum fluorescence yield of PSII photochemical reaction expressed as ratio of variable fluorescence (F_M_ − F_0_) and maximum fluorescence, calculated according to the equation:
  $$ {{{\text{F}}_{{\text{V}}} } \mathord{\left/ {\vphantom {{{\text{F}}_{{\text{V}}} } {{\text{F}}_{{\text{M}}} }}} \right. \kern-\nulldelimiterspace} {{\text{F}}_{{\text{M}}} }} = {{\left( {{\text{F}}_{{\text{M}}} - {\text{F}}_{0} } \right)} \mathord{\left/ {\vphantom {{\left( {{\text{F}}_{{\text{M}}} - {\text{F}}_{0} } \right)} {{\text{F}}_{{\text{M}}} }}} \right. \kern-\nulldelimiterspace} {{\text{F}}_{{\text{M}}} }} $$

where F_V_ represents the maximum variable fluorescence calculated as F_M_ − F_0_, F_M_ corresponds to the maximum fluorescence emission and F_0_ is the minimum fluorescence emission.

It reflects the efficiency of PSII in using light for photochemical conversion and its value is usually at 0.8 in physiological conditions or decreased values under stress.

Furthermore, J and I intermediate points represents a direct indication of transient changes in the plastoquinone redox state within the PSII reaction centre, as the accumulation of Q_A_ in reduced state and the consequent filling up of the plastoquinone pool with electrons. Any change in the PSII electron transfer efficiency directly causes variations in the fluorescence intensity of the inflection points, as a consequence of different stress conditions. In particular, the variable fluorescence parameter 1 − V_J_ provides a specific response directly related to the herbicide-D1 binding niche interaction, and it can be calculated according to the equation:

$$ 1 - {\text{V}}_{{\text{J}}} = 1 - {{\left( {{\text{F}}_{{{\text{2ms}}}} { - }{\text{F}}_{{0}} } \right)} \mathord{\left/ {\vphantom {{\left( {{\text{F}}_{{{\text{2ms}}}} { - }{\text{F}}_{{0}} } \right)} {\left( {{\text{F}}_{{\text{M}}} { - }{\text{F}}_{{0}} } \right)}}} \right. \kern-\nulldelimiterspace} {\left( {{\text{F}}_{{\text{M}}} { - }{\text{F}}_{{0}} } \right)}} $$

where F_0_, F_M_, F_2ms_ are respectively the initial fluorescence, the maximum fluorescence and the fluorescence measured after 2 ms [15].

### Algae immobilisation protocol

*Chlamydomonas reinhardtii* cell cultures in early mid-exponential growth phase, with Abs_750_ 0.7 O.D., 10^7^ cells/mL, and 10 µg/mL chlorophyll content, were exploited for the immobilisation on paper-based screen-printed electrodes nanomodified with carbon black (pCB-SPEs). A volume of 14 mL of cell cultures were harvested by 10 min centrifugation at 2000×*g* and 15 °C to obtain a final amount of cells equal to 1.2 × 10^7^. The cell pellet was re-suspended in 50 µL of 50 mM tricine pH 7.2, and mixed with 100 μl of a 1% (w/v) sodium alginate solution in the same buffer, obtaining a final cell concentration of 0.08 × 10^6^ cells/L. 5 μl of this suspension, containing ~ 4 × 10^5^ cells, were deposited over a carbon black nanomodified working electrode surface (diameter 3.0 mm) of a three-electrodes system printed on paper, with a carbon and an Ag/AgCl reference electrode. 5 μl of 200 mM CaCl_2_ in 50 mM Tricine-NaOH were drop cast on the deposited algae to allow the physical gelation of the alginate and the consequent entrapment of cells on the working electrode, keeping in mind that this step needs to be done more quickly to avoid damage to the algae cells due to running dry. Finally, algae/pCB-SPEs were stored into 50 mM Tricine, 20 mM CaCl_2_, 5 mM MgCl_2_, 50 mM NaCl, 70 mM sucrose pH 7.2 and incubated for 2 h under continuous light (50 µmol photons m^−2^ s^−1^) and 25 °C.

### Herbicide analysis

The optical and electrochemical analysis of the selected herbicides was provided by following both the variable fluorescence parameter 1 − V_J_ and the oxygen evolution capacity, respectively, using a dual electro-optical transducer prototype (Biosensor srl, Via degli Olmetti, Rome, Italy). Algae were illuminated by a 350 µmol photons m^−2^ s^−1^ light with repeated cycles of 30 s light excitation and 10 min dark. An applied potential of − 0.6 V was used with an acquirement interval of 0.5 s. Herbicides were added into the electrochemical cell (200 µL volume containing 50 mM Tricine, 20 mM CaCl_2_, 5 mM MgCl_2_, 50 mM NaCl, 70 mM sucrose pH 7.2) in a concentration range from 1 to 100 µM and the 1 − V_J_ variations as well as the current signals due to oxygen reduction on the pCB-SPE working electrode were recorded in dependence to the target analyte concentrations.

### Biosensor prototypes

**Fig. 1:**
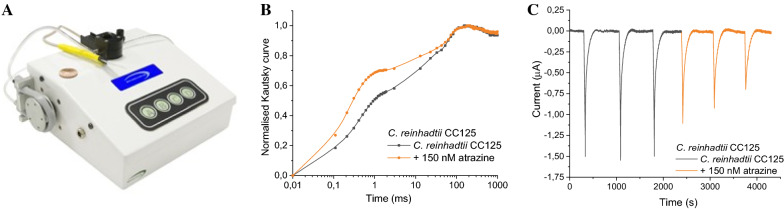
Dual electro-optical transducer prototype (**A**) for the measurement of the chlorophyll *a* fluorescence (**B**) and for the measurement of the current signals of algae under light illumination (**C**)

The paper-based screen-printed electrode algae sensors were integrated into the dual electro-optical transduction prototype able of both optical and electrochemical analysis, projected and realised by the company Biosensor srl. The instrument is a portable prototype consisting of a module cell for the insertion of the paper-based screen-printed electrode hosting the biological material. The cell is equipped with a LED system (of 350 μmol photons m^−2^ s^−1^ of red light at a 650 nm wavelength) which provide algae illumination and measure the fluorescence emission at 680 nm by a photodiode, automatically calculating the fluorescence parameters (F_V_/F_M_ and 1 − V_J_) according to the recorded Kautsky curves (Fig. 1B). The red light covers a surface of 1 cm^2^, resulting in highly reproducible measurements averaged over 1000 points. The electrochemical set-up is constituted of a DC voltage supply, which provide a bias potential in the range of ± 0.800 V between the working and the reference electrodes, and an amperometer to detect the current intensity variation deriving from the algae oxygen evolution process (Fig. 1C). The biological module, perfectly sealed, hosts the samples under test. Both static and dynamic operations are allowed thanks to an automatically controlled fluidic system equipped of inlet and outlet connections for the electrolytic/washing solution and sample flow.

## Results and discussions

### A systematic characterisation of 28 strains of *C. reinhardtii*

With the aim to increase the selectivity of algae-based biosensors we propose a systematic study of a collection of 28 strains of the green alga *C. reinhardtii* to exploit as an array of biocomponent with different affinity towards diverse herbicide classes. The intention is to provide a multiplexing analysis of target analytes of agro-environmental interest by the evaluation of inhibition studies with solutions and mixtures of herbicides.

The choice of the bioreceptor during biosensor design is an essential phase, which deserves the right assessments in terms of sensitivity, repeatability, and stability among other analytical parameters. To meet these demands, many inspirations have been originated by docking and molecular genetics approaches, to obtain very sensitive and selective bioreceptors. In the last decades, more than 50 different *C. reinhardtii* algae strains have been collected in our laboratories and used for different purposes (e.g., nutraceutical, space research, and biosensing) [14]. This handbook on biosensor design started from the selection of 28 different strains of *C. reinhardtii* from this collection, obtained in previous studies by diverse techniques as cloning, mutagenesis, and biolistic transformation. Among them, CC125 is the wild type strain exploited as control [12]. This corresponds to the basic “137c” wild type (isolated in 1945 near Amherst MA, USA, by G.M. Smith) and is presumably equivalent to strain 11/32c (Culture Centre of Algae and Protozoa, location, country). CC125 is the background strain for many mutations, including CC503 cw92 mt+, which has been the source of DNA, used for genomic sequencing. CC125 has been kindly provided by the Chlamydomonas Collection, Duke University to UTEX in 1980 (UTEX 2244).

CW15 is a cell-wall mutant with a particular defect in cell wall production [11, 20].

The intron less (IL) strain has been produced from wild-type cells 11/32b using an efficient chloroplast transformation protocol. In particular, the highly conserved *psbA* gene has been manipulated, and a transformant with no introns in the *psbA* gene has been generated starting from wild-type cells 11/32b (Sammlung von Algenkulturen, Grttingen, FRG). Starting from this strain, the deletion mutant FuD7 and the Del mutant have been obtained in previous studies as well as a collection of different mutants [17]. For this reason, IL is considered as a reference strain for the 25 D1 mutants selected for this protocol and reported in Table 1.

**Table 1:** List of the D1 site directed mutants selected for the systematic characterisation of potential biorecognition element

Site directed mutants	WT	Mutant	Hydropathic index/reactivity class/side chain polarity				Localization of the mutation in the protein D1
			WT		Mutant		
A153S	Alanine	Serine	1.8 (II)	Nonpolar	− 0.8 (0)	Polar	In the helix iii of d1
A250L	Alanine	Leucine	1.8 (II)	Nonpolar	3.8 (IV)	Nonpolar	In the stroma loop ii
A250R	Alanine	Arginine	1.8 (II)	Nonpolar	− 4.5 (0)	Polar	In the stroma loop ii
A250V	Alanine	Valine	1.8 (II)	Nonpolar	4.2 (III)	Nonpolar	In the stroma loop ii
A251C	Alanine	Cysteine	1.8 (II)	Nonpolar	2.5 (V)	Polar	In the stroma loop ii
F182M/I184M	Phenylalanine	Methionine	2.8 (VI)	Nonpolar	1.9 (V)	Nonpolar	In the helix v of d1
	Isoleucine	Methionine	4.5 (IV)	Nonpolar	1.9 (V)	Nonpolar	
F255N	Phenylalanine	Asparagine	2.8 (VI)	Nonpolar	− 3.5 (0)	Polar	In the stroma loop ii
F265S	Phenylalanine	Serine	2.8 (VI)	Nonpolar	− 0.8 (0)	Polar	In the ii stroma loop
F265T	Phenylalanine	Threonine	2.8 (VI)	Nonpolar	− 0.7 (0)	Polar	In the ii stroma loop
G207S	Glycine	Serine	− 0.4 (I)	Nonpolar	− 0.8 (0)	Polar	In the helix iv of d1
L159I	Leucine	Isoleucine	3.8 (IV)	Nonpolar	4.5 (IV)	Nonpolar	Near to tyr 161
L159V	Leucine	Valine	3.8 (IV)	Nonpolar	4.2 (III)	Nonpolar	Near to tyr 161
L159M	Leucine	Methionine	3.8 (IV)	Nonpolar	1.9 (V)	Nonpolar	Near to tyr 161
L200I	Leucine	Isoleucine	3.8 (IV)	Nonpolar	4.5 (IV)	Nonpolar	In the helix iv of d1
I163F	Isoleucine	Phenylalanine	4.5 (IV)	Nonpolar	2.8 (VI)	Nonpolar	Near to tyr 161
I163N	Isoleucine	Asparagine	4.5 (IV)	Nonpolar	− 3.5 (0)	Polar	Near to tyr 161
I163S	Isoleucine	Serine	4.5 (IV)	Nonpolar	− 0.8 (0)	Polar	Near to tyr 161
I163T	Isoleucine	Threonine	4.5 (IV)	Nonpolar	− 0.7 (0)	Polar	Near to tyr 161
I281T	Isoleucine	Threonine	4.5 (IV)	Nonpolar	− 0.7 (0)	Polar	In the helix v of d1
M172L	Methionine	Leucine	1.9 (V)	Nonpolar	3.8 (IV)	Nonpolar	Near to oec
P162S	Proline	Serine	− 1.6 (III)	Nonpolar	− 0.8 (0)	Polar	Near to tyr 161
S212C	Serine	Cysteine	− 0.8 (0)	Polar	2.5 (V)	Polar	In the helix iv of d1
S264K	Serine	Lysine	− 0.8 (0)	Polar	− 3.9 (0)	Polar	In the stroma loop ii
S268C	Serine	Cysteine	− 0.8 (0)	Polar	2.5 (V)	Polar	In the stroma loop ii
S209A/S212C	Serine	Alanine	− 0.8 (0)	Polar	1.8 (II)	Nonpolar	In the helix iv of d1
	Serine	Cysteine	− 0.8 (0)	Polar	2.5 (V)	Polar	

The amino acidic substitution and properties are indicated, as well as mutation position respect the D1 protein structure. The hydropathic index is cited as in Kyte et al. [19]; the amino acid classification in reactivity classes is according to the increase reactivity towards reactive oxygen species, and is done according to Cooper et al. [10], the side chain polarity is presented according to Rea et al. [22]

The selected 28 strains were physiologically characterised, by evaluating cell culture growth, pigment content, photosynthetic activity, oxygen evolution, and herbicide detection capability, with the aim to exploit them as bioreceptors for the realisation of a dual electro-optical transduction algal biosensor.

#### Cell culture growth

**Fig. 2:**
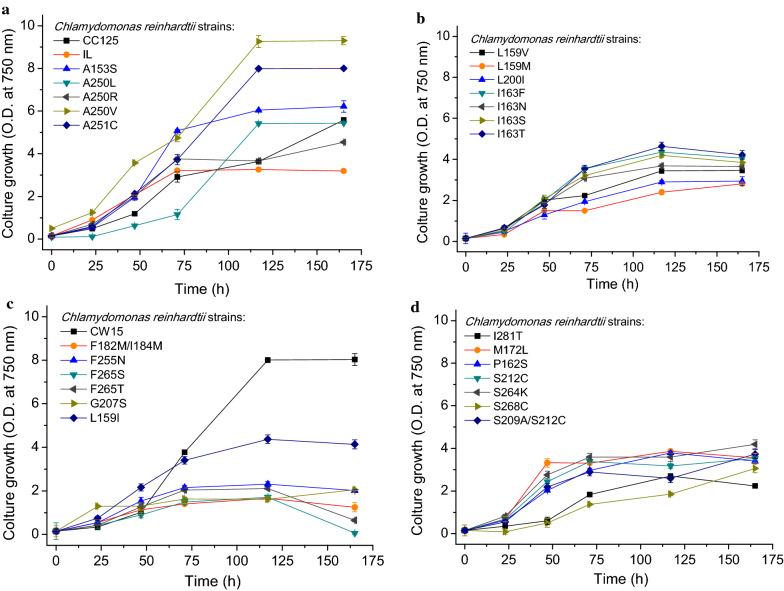
Growth curve of *C. reinhardtii* 28 strains reported in Table 1, measuring the optical density (O.D.) of the cell suspension at 750 nm wavelength. Cells are growth in TAP at 25 °C under continuous light 50 µmol photons m^−2^ s^−1^ and continuous stirring at 150 rpm (n = 3)

The growth of selected *Chlamydomonas* strains was characterized by measuring the optical density of the culture over a 165-h period at a 750 nm wavelength. Figure 2 reports the time courses of all the 28-strain culture growth, showing the difference among the grow rate of D1 mutants, the reference strain IL, the wild type control CC125, and the mutant cw15. As shown, during the analysed period, absorbance values were found similar for most D1 mutants, and slightly higher for A153S, A250V, A251C, probably due to the particular amino acidic substitution, as well as for the wild type CC125 and cw15 mutant. Values are the average of 3 independent experiments (± SE, n = 3).

#### Pigment content, photosynthetic activity, and oxygen evolution

**Fig. 3:**
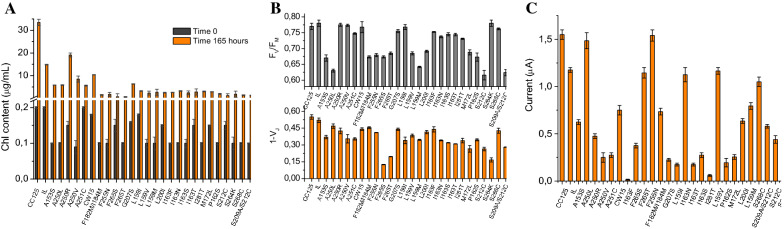
**A** *C. reinhardtii* 28 strains pigment content calculated on the basis of the chlorophyll extract optical density (O.D.) at 652 nm wavelength. **B** F_V_/F_M_ and 1 − V_J_ values. **C** Oxygen evolution measured by CB-SPEs. Cell cultures at early mid-exponential phase (Abs_750_ = 0.5 O.D., 10^6^ cells/mL, and 5 µg/mL chlorophyll content) grown under 50 μmol m^−2^ s^−1^ continuous light at 25 °C and continuous stirring at 150 rpm (n = 3)

The pigment content of selected *Chlamydomonas* strains was analysed by following the chlorophyll *a* and *b* accumulation of the cultures over a 165-h period under continuous light (50 µmol photons m^−2^ s^−1^), agitation (150 rpm) and 25 °C temperature, obtained by 80% acetone extraction and spectrophotometrically measured as absorbance at a 652 nm wavelength. As depicted in Fig. 3A, the chlorophyll content in the wild type CC125 was, in the majority of cases, more than three times as high as in most mutants, excluding IL and A250R. The photochemical efficiency of *C. reinhardtii* strains was evaluated by the fluorescence analysis of the chlorophyll *a* described by the OJIP or Kautsky profile *vs* time, by which it is possible to calculate the characteristic photosynthetic parameters F_V_/F_M_ (maximum fluorescence yield of PSII) and 1 − V_J_ (relative variable fluorescence at 2 ms). The values of these fluorescence parameters were reported in Fig. 3B, calculated by each Kautsky curve and plotted as a function of time, to show the photosynthetic efficiency of the different strains in physiological conditions. These results evidence that some strains (e.g., CC125, IL, A250R, A250V, CW15, S264K) exhibit a higher photosynthetic activity in terms of maximum fluorescence yield in comparison with other strains (e.g., A153S, A250L, L159M, S212C among others) which show a reduction of this parameter. On the contrary, some strains, with a sufficient maximum fluorescence yield, show decreased values of the variable fluorescence parameter, as for example the strain S264K. In general, the variations of the photosynthetic parameters could be ascribed to a rearrangement of the photosynthetic apparatus due to the induced mutations in the D1 protein, as well as to a change in electron transfer rate along with the photosynthetic chain, in particular between the first electron acceptor Q_A_ and the second one Q_B_. As a result, the evident variation in shape of the OJIP curve of these mutants with respect to the control strain is directly attributable to the accumulation of reduced Q_A_ and to the marked slowing of electron flow.

Oxygen evolution for the 28 *C. reinhardtii* strains was amperometrically measured on liquid cell cultures exploiting carbon black (CB) nanomodified screen-printed electrodes (SPEs) as a valid alternative to the conventional Clark electrode. In detail, carbon black nanoparticles provide the surface for the reduction of oxygen at an applied potential of − 0.6 V. This enables an increasing of the sensitivity and the shifting of the applied potentials to lower ones, thus avoiding interfering species occurring at higher potentials. In our previous work, we demonstrated the effectiveness of using CB-SPEs for oxygen detection in terms of improved sensitivity and decreased applied potential, in comparison with bare electrodes as well as with multi-walled carbon nanotube-SPE [8]. Indeed, CB-SPEs sense oxygen reduction thanks to their ability to electrocatalyse the reduction of oxygen at a lower potential than unmodified SPEs, with an increase of sensitivity due to nanodimensions onion-like carbon structure, high number of defect sites, and higher content in O atoms [3–6, 21, 27]. When compared with other carbon nanomaterials including carbon nanotubes and graphene, CB is further characterised by cost-effectiveness, easiness to prepare a stable dispersion for SPE modification by drop-casting procedure, and the absence of chemical treatment before use, rendering it a most sustainable choice. In this case, CB is able to measure oxygen produced within the photosynthetic process when algae are under illumination (350 µmol photons m^−2^ s^−1^). The rate of oxygen evolution was measured for each strain at an applied potential of − 0.6 V after a period of 10 min of dark adaptation. As shown in Fig. 3C, CC125, A250L and F255N strains exhibited the highest current signals up to 1.5 µA.

#### Herbicide detection capability

**Fig. 4:**
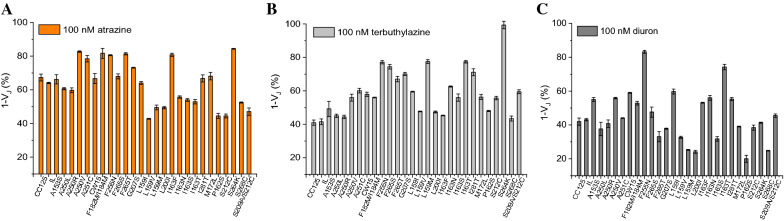
Response of the 28 *C. reinhardtii* strains reported in Table 1 to a 100 nM concentration of atrazine (**A**), terbuthylazine (**B**), and diuron (**C**) (n = 3). Cell cultures at early mid-exponential phase (Abs_750_ = 0.5 O.D., 10^6^ cells/mL, and 5 µg/mL chlorophyll content) grown under 50 μmol m^−2^ s^−1^ continuous light at 25 °C and continuous stirring at 150 rpm (n = 3)

The response of the selected strains to triazine and urea type herbicides was studied by chlorophyll *a* fluorescence induction curves (OJIP or Kautsky curve). The relative variable fluorescence at 2 ms at the characteristic point J (V_J_) gives a measure of the relative amount of the reduced Q_A_. Therefore, 1 − V_J_ represents the fraction of oxidised Q_A_ or the efficiency by which the electron is transferred from plastoquinone Q_A_ to Q_B_. In the presence of photosynthetic herbicides, the Q_B_ binding site is permanently occupied by the inhibitor and electron transport does not extend beyond Q_A_. The accumulation of reduced forms of Q_A_ is proportional to the herbicide concentration and can be directly evaluated by the increase of the variable fluorescence level at point J, making the parameter 1 − V_J_ one of the most sensitive for herbicide detection. Using this approach, we evaluate the sensitivity of the selected strains to atrazine, terbuthylazine, and diuron as case study herbicides. Additional file 1: Figure S1 reports the fluorescence response of the 28 *Chlamydomonas* strains towards the three herbicides as Kautsky curves as well as calibration curves expressed as 1 − V_J_ vs herbicide concentration. Moreover, with the aim to highlight the different sensitivity of all the strains, their response to a fixed herbicide concentration of 100 nM is reported in Fig. 4, which underline how each strain demonstrates different inhibition of the photosynthetic activity in the presence of the herbicides.

Among other, L159V, L159M, L200I, I163N, I163S, I163T, P162S, S212C, S268C strains and the double mutant S209A/S212C showed the highest sensitivity to atrazine with a ~ 50% of photosynthetic activity inhibition. While L159V presented the highest sensitivity to atrazine, CC125, IL, and S268C showed the highest sensitivity to terbuthylazine (~ 50% inhibition). Whereas, S264K strain is resistant to triazine while very sensitive to urea herbicides. In addition, most strains are highly susceptible to diuron exposure, with an inhibition from 60% (e.g., for CC125, IL, A250L, A250R, and A251C) to 80% (for L159M, L200I, P162S, S268C). Notably, F255N strain showed high resistance to both herbicide classes.

The inhibition constant IC_20_ (herbicide concentration required for 20% inhibition of the parameter 1 − V_J_) and detection limits were also calculated (Table 2) on the basis of 99% confidence interval, which, assuming the normal distribution of the data, corresponds to 2.6 × standard error of the measurements (σ), exploiting the modified relationship for the Langmuir absorption isotherm, ${\text{LOD}}\, = \,2.6\, \times \,{\upsigma }\, \times \,{\text{IC}}_{20} /(100 - 2.6\, \times \,{\upsigma })$ [18].

**Table 2:** Limits of detection of *C. reinhardtii* selected strains to three different photosynthetic herbicides

	Atrazine	Terbuthylazine	Diuron
	LOD pM	LOD pM	LOD pM
CC125	33	32	32
IL	32	29	23
A153S	46	23	35
A250L	45	15	28
A250R	23	26	26
A250V	***R***	27	40
A251C	60	41	31
CW15	47	33	47
F182M/I184M	***R***	***R***	27
F255N	***R***	84	14
F265S	41	86	17
F265T	***R***	46	14
G207S	79	79	21
L159I	16	26	21
L159V	15	20	13
L159M	33	73	18
L200I	21	14	13
I163F	***R***	26	29
I163N	17	17	14
I163S	21	27	15
I163T	14	26	27
I281T	25	25	14
M172L	31	25	23
P162S	15	14	13
S212C	32	26	15
S264K	***R***	14	18
S268C	47	26	14
S209A/S212C	29	45	25

***R*** resistant

### *Chlamydomonas reinhardtii* immobilisation on paper-based SPEs

Once assessed the physiological features of the selected strains, further experiments for the development of the dual electro-optical biosensor have been accomplished exploiting *C. reinhardtii* CC125 strain as a case study.

A limiting step in the development of whole cell biosensors is the immobilization of the biomaterial in a matrix that prevents leaching, without reducing the stability and activity of the cells. Several methods are currently available for cell immobilisation, with entrapment as the most frequently used. With the scope to enhance the yield in the production of working algae-sensors as well as to achieve a higher repeatability in algae-sensor preparation, algal whole cells were immobilised on paper-based screen-printed electrodes modified with carbon black nanomaterials (paper-SPEs). The rationale behind the choice of using electrodes printed on paper consists of capturing algal whole cells into a 3D matrix composed of a natural polymer, thus providing a comfortable environment for cells accommodation guaranteeing a better algae survival as well as an effective diffusion across the matrix of the target analyte.

**Fig. 5:**
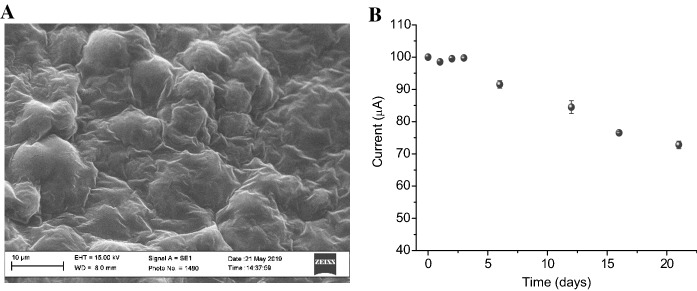
**A** SEM analysis of the paper-SPEs with immobilised CC125 *C. reinhardtii*. **B** Storage stability expressed as current signals of algae-paper-SPE sensors stored at 25 °C under light illumination at 50 μmol photons m^−2^ s^−1^ (n = 3)

To this aim, whole cells of *C. reinhardtii* CC125 were immobilised on the working electrode surface of paper-based screen-printed electrodes nanomodified with carbon black (paper-SPEs). To preserve the algae photosynthetic functionality, cells were entrapped in a calcium/alginate matrix. Thus, 1.2 × 10^7^ cells of CC125 are suspended in 50 L of 1% sodium alginate and 100 L of 50 mM Tricine, 20 mM CaCl_2_, 5 mM MgCl_2_, 70 mM sucrose pH 7.2. Then, 5 L of this suspension were drop cast on the working electrode of the SPE and then cross-linked using 5 L of 200 mM CaCl_2_ in the same buffer, obtaining a final number of cells of 4 × 10^5^. This calcium alginate matrix can provide a biocompatible environment for algae entrapment, without collateral effect on their metabolism; at the same time, its porous network guarantees a good diffusion of the target analyte. The algae/pCB-SPEs were thus stored in 200 mM CaCl_2_ in 50 mM Tricine, 20 mM CaCl_2_, 5 mM MgCl_2_, 70 mM sucrose pH 7.2 under continuous light at 50 μmol photons m^−2^ s^−1^ for successive measurements. Each algae/paper-SPE was then accommodated into the measurement cells of the electro-optical transducer for the electrochemical analysis. SEM analysis and storage stability were performed to characterise the implemented algae-SPE sensors in depth (Fig. 5A, B; respectively).

### Study on different (nano)modified SPEs and working stability

**Fig. 6:**
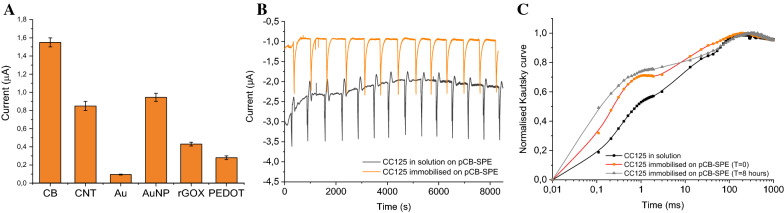
**A** Current signal from oxygen evolution of CC125 *C. reinhardtii* whole cells immobilised on paper-based screen-printed electrodes modified with different (nano)materials. Working stability of *C. reinhardtii* immobilised on pCB-SPEs evaluated by amperometric (**B**) and fluorescence (**C**) analysis. Applied potential − 0.6 V, interval analysis time 0.5 s, repeated cycles of 10 min dark and 30 s light (350 μmol photons m^−2^ s^−1^) (n = 3)

Different screen-printed electrodes modified with diverse (nano)materials were exploited to evaluate the best material for the analysis of the current signals coming from the algae oxygen evolution, including carbon black, carbon nanotubes, gold, gold nanoparticles, reduced graphene oxide, and poly(3,4-ethylenedioxythiophene) (PEDOT). Results reported in Fig. 6A highlight how carbon black represents the best choice, as this nanomaterial is able to enhance the electron transfer between algae evolved oxygen and the working electrodes, thus amplifying the current signals, especially in comparison with other (nano)materials.

Once selected the best nanomodified SPE, working stability of CC125 *C. reinhardtii* in solution and immobilised on paper-based SPEs modified with carbon black (pCB-SPEs) was evaluated by both amperometric and fluorescence analysis, run for many hours at room temperature, under repeated cycles of 10 min dark and 30 s light (350 μmol photons m^−2^ s^−1^) in a measurement volume of 200 L of 50 mM Tricine, 20 mM CaCl_2_, 5 mM MgCl_2_, 50 mM NaCl, 70 mM sucrose pH 7.2. Figure 6B shows 100% of light-induced oxygen evolution activity up to ca. 8–10 h, with a progressive decrease of signal later on. This indicates not only a very good operational stability but also a high intra-electrode repeatability with RSD of 1.1% (n = 12). However, there is a clear difference between the signal stability of algae in solution and immobilised, evidencing that the immobilisation confers a higher robustness of the sensor. Figure 6C reports the Kautsky profiles of algae in solution as well as immobilised, underlining that the algae photosynthetic activity slightly suffers from the immobilisation while remaining constant during the 8–10 h of the stability test.

### Reversibility, matrix effect, and interferents

**Fig. 7:**
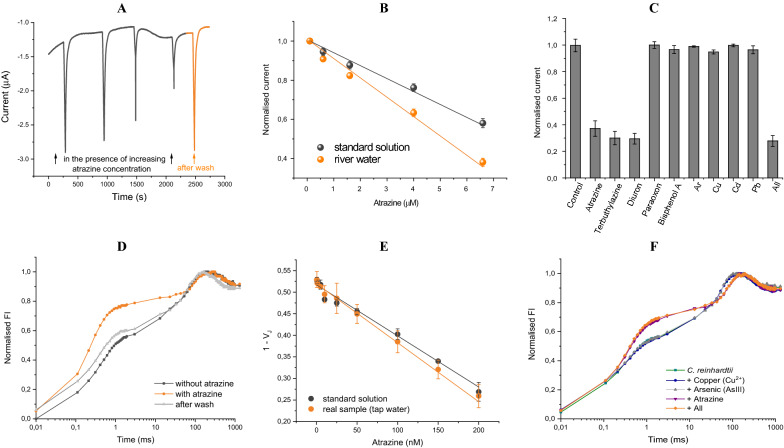
CC125 *C. reinhardtii* reversibility tests by amperometric (**A**) and fluorescence (**D**) analysis. Matrix effect by amperometric (**B**) and fluorescence (**E**) analysis. Study of the interferents by amperometric (**C**) and fluorescence (**F**) analysis. Applied potential − 0.6 V, interval analysis time 0.5 s, repeated cycles of 10 min dark and 30 s light (350 μmol photons m^−2^ s^−1^) (n = 3)

To gain further insights on the evaluation of the diverse analytical parameters, tests were performed to evaluate the nature of herbicide inhibition by both amperometric (Fig. 7A) and fluorescence analysis (Fig. 7D), exploiting atrazine as case study. These tests consist in washing the algae/pCB-SPE with 50 mM Tricine, 20 mM CaCl_2_, 5 mM MgCl_2_, 50 mM NaCl, 70 mM sucrose pH 7.2 after the measurement of the inhibition at an atrazine concentration of 6.6 M, for the electrochemical analysis, and 100 nM, for the optical one, to record both the recovered current and fluorescence signals. As reported in Fig. 7A, D, a complete recovery of both algae oxygen evolution and photosynthetic activity occurred.

To investigate the suitability of the proposed biosensor in real samples, matrix effect and recovery studies were also provided. To estimate matrix effect by the electrochemical point of view, algae/pCB-SPEs were incubated in 200 L of 2 × buffer (100 mM Tricine, 20 mM CaCl_2_, 5 mM MgCl_2_, 50 mM NaCl, 70 mM sucrose pH 7.2) diluted 1:2 (v:v) in surface water (Sebou River, Morocco) and fortified with atrazine in a concentration range from 0.1 to 41.6 μM. Calibration curves were obtained for both standard solutions and real samples, described by the equations y = 0.98(± 0.01) − 0.06 (± 0.003) × (R^2^ = 0.9952) and y = 0.98 (± 0.01) − 0.09 (± 0.005) × (R^2^ = 0.9945), respectively (Fig. 7B). The biosensor response was in the linear range from 0.1 to 6.6 μM atrazine and showed a detection limit of 2 nM. The ratio between slopes of the calibration curves obtained in standard solutions and real samples was equal to 0.72, indicating a moderate dependence from surface water matrix (28%). The calibration curve obtained for the electrochemical algal biosensor challenged in real samples was further used to calculate the recovery values of the surface water samples. Recovery values of 106.6 ± 10% and 96 ± 8% were obtained for 3 and 5 M of atrazine, respectively, highlighting a satisfactory capability to detect atrazine also in surface water.

Regarding the fluorescence analysis, tap water was exploited as real environmental matrix fortified with atrazine in a nanomolar concentration range. Calibration curves were obtained for both standard solutions and real samples, described by the equations y = 0.516 (± 0.005) − 0.0012 (± 0.00006) × (R^2^ = 0.9854) and y = 0.518 (± 0.003) − 0.0013 (± 0.00003) × (R^2^ = 0.9963), respectively (Fig. 7E). The biosensor response was in the linear range from 10 to 200 nM atrazine and showed a detection limit of 5 pM. The slopes of the calibration curves obtained in standard solutions and real samples were very similar with a ratio of 0.92, indicating a slight dependence from the tap water matrix. The calibration curve obtained for the optical transduction challenged in real samples was further used to calculate the recovery values of the tap water samples. A recovery value of 96 ± 5% was obtained for 75 nM of atrazine, highlighting a satisfactory capability to detect atrazine also in tap water.

It is crucial to point out the observation of the different detection limits obtained for the two detection methods exploited, highlighting that the optical transduction provides atrazine analysis in the nanomolar range while the electrochemical one in the micromolar range.

With the aim to challenge the capability of the proposed algal biosensor in real environmental water samples, some chemicals including metals, pesticides, and phenolic compounds were tested as interferences at legal limits established by the EU Directive 2013/39/EU for surface water (where present). In detail, algae/pCB-SPEs were incubated in 200 L of 50 mM Tricine, 20 mM CaCl_2_, 5 mM MgCl_2_, 50 mM NaCl, 70 mM sucrose pH 7.2 fortified with standard solutions of 100 ppb arsenic, 20 ppb copper, 5 ppb cadmium, 10 ppb lead, 10 ppb bisphenol A, 1 ppb paraoxon, and 6.6 M atrazine, terbuthylazine, and diuron, as well as with a solution spiked with all the above listed compounds, to evaluate the synergistic effect of different chemicals in a mixture. Results reported in Fig. 7C, F highlight that the interfering species did not affect the analysis of atrazine at the exploited concentrations in both electrochemical and fluorescence analysis.

## Conclusions

Main issues related to the development of algal-based biosensors were addressed and mitigated in this study. In particular, the exploitation of a multi-array platform boosted to increase the selectivity, up to now referred to as a main concern [16]. First attempts have already been made in previous works, in which the multi-selectivity of the platform was due to the presence of biorecognition elements from different sources (both enzymes and algae cells) towards diverse targets [23]. For the first time, here we focus on the exclusive use of algae whole cells, proposing the design of an array platform of bioreceptors for the simultaneous detection of different classes of herbicides by a systematic study of a *C. reinhardtii* algae collection. The concept of using the array of algae strains relies on the difference in the response behaviour of the individual bioreceptor towards diverse herbicide classes; therefore, it was interesting to compare the inhibition results of all the 28 strains to get differentiated information about the presence of pollutants or groups of them.

Another important drawback hindering the success of algae-based biosensors is represented by the low repeatability of the algae-sensor fabrication, strictly related to cells preparation and immobilization on the selected surface, e.g., filter paper and screen-printed electrodes. For this reason, many amendments on protocols, already reported in the literature on this topic, are made, and positive feedbacks obtained as shown by the experimental data above reported. The first delicate step is the harvesting of *C. reinhardtii* cells. In the present study, the conditions of algae centrifugation were modified by lowering speed from 5000 to 2000*g* as well as reducing centrifugation time from 10 to 5 min, making this preparation step much softer and less destructive for the cells. In addition, number of cells (from 10^6^ to 10^7^ cells/ml), and content of chlorophylls (from 8 to 10 µg/ml) were increased to enrich the biological material to be immobilised.

Furthermore, the addition of alginate and calcium chloride represents another important critical aspect, and reducing the concentration of alginate from 2 to 1% (w/v) allows to improve algae performance and thus to increase the repeatability of sensor preparation. Indeed, these new favourable conditions furnished a suitable behaviour and thus a better vitality of the cells, and, at the same time, a less dense porous network guaranteed a good diffusion of the target analytes. This led to a higher repeatability in algae-sensor preparation with RSD of 5% (n = 100) in comparison with previous results.

In this overall scenario, the proposed biosensor shows many advantages in comparison with most photosynthetic biosensing systems available in literature, in terms of sensitivity, repeatability, working and storage stability, and capability to operate in real water matrices.

On the other hand, a main challenge emerged from this immobilisation protocol, that is cell leaching from the supports, especially in the case of filter paper. Indeed, despite significant advantages of sustainability, affordability and effectiveness of paper-based algae sensors, they can actually be exploited only for static measurements. In fact, leaching phenomenon occurs in dynamic analysis, probably due to a weak cells adhesion on the support. This will commit our group to carry out new trials, aimed at developing a new immobilisation protocol devoted to measures in fluidics. In this context, further perspectives can be addressed towards the development of sandwich-type polymeric films for a stronger embedding of algae cells into the support, eventually also constituted of nanomaterials (i.e., graphene) for the improvement of both cell adhesion and photosynthetic/analytical performances.

## Supplementary Information

**Additional file 1: Figure S1.** Kautsky profiles and calibration curves of the 28 C. *reinhardtii* strains in the presence of atrazine, terbuthylazine, and diuron.

## Data Availability

All data generated or analysed during this study are included in this published article within each editable graph.
