# Supplementary material for: A dual electro-optical biosensor based on Chlamydomonas reinhardtii immobilised on paper-based nanomodified screen-printed electrodes for herbicide monitoring
Source: J Nanobiotechnology. 2021 May 17;19:145. doi: 10.1186/s12951-021-00887-4 (PMC8130446; doi:10.1186/s12951-021-00887-4)
Supplement: Supplementary file 1 — Additional file 1: Figure S1. Kautsky profiles and calibration curves of the 28 C. reinhardtii strains in the presence of atrazine, terbuthylazine, and diuron. [file 12951_2021_887_MOESM1_ESM.docx]

| **Strain** | **Atrazine** | **Terbuthylazine** | **Diuron** | **Calibration curves** |
| --- | --- | --- | --- | --- |
| **CC125** |  |  |  |  |
| **IL** |  |  |  |  |
| **A153S**   |  |  |  |  |
| **A250L** |  |  |  |  |
| **A250R** |  |  |  |  |
| **A250V** |  |  |  |  |
| **A251C** |  |  |  |  |
| **CW15** |  |  |  |  |
| **F182M/I184M** |  |  |  |  |
| **F255N** |  |  |  |  |
| **F265S** |  |  |  |  |
| **F265T** |  |  |  |  |
| **G207S** |  |  |  |  |
| **L159I** |  |  |  |  |
| **L159V** |  |  |  |  |
| **L159M** |  |  |  |  |
| **L200I** |  |  |  |  |
| **I163F** |  |  |  |  |
| **I163N** |  |  |  |  |
| **I163S**   |  |  |  |  |
| **I163T**   |  |  |  |  |
| **I281T** |  |  |  |  |
| **M172L** |  |  |  |  |
| **P162S** |  |  |  |  |
| **S212C** |  |  |  |  |
| **S264K** |  |  |  |  |
| **S268C** |  |  |  |  |
| **S209A/S212C** |  |  |  |  |

Figure S1.
